# Supplementary material for: Mobile phone-based interventions for mental health: A systematic meta-review of 14 meta-analyses of randomized controlled trials
Source: PLOS Digit Health. 2022 Jan 18;1(1):e0000002. doi: 10.1371/journal.pdig.0000002 (PMC8881800; doi:10.1371/journal.pdig.0000002)
Supplement: S1 Table — (DOCX) [file pdig.0000002.s001.docx]

**Supplemental Materials Table S1**. Cochrane risk of bias assessment at the meta-analysis level

|  | Rand Seq | | | Allocat Conceal | | | Blind Person/Partic | | | Blind Outcome | | | Incomp Data | | | Select Report | | | Other | | |
| --- | --- | --- | --- | --- | --- | --- | --- | --- | --- | --- | --- | --- | --- | --- | --- | --- | --- | --- | --- | --- | --- |
| Meta-analysis | + | ? | - | + | ? | - | + | ? | - | + | ? | - | + | ? | - | + | ? | - | + | ? | - |
| Cox (2020) | 56 | 22 | 22 | 11 | 89 | 0 | 0 | 0 | 100 | 33 | 33 | 33 | 33 | 22 | 44 | 11 | 78 | 11 | 56 | 33 | 11 |
| Firth (2017a) | 56 | 44 | 0 | 56 | 44 | 0 | 11 | 56 | 33 | 78 | 22 | 0 | 100 | 0 | 0 | 100 | 0 | 0 | 44 | 22 | 33 |
| Firth (2017b) | 83 | 17 | 0 | 72 | 22 | 6 | 28 | 28 | 44 | 67 | 22 | 11 | 83 | 0 | 17 | 100 | 0 | 0 | 39 | 28 | 33 |
| Gál (2021) | 65 | 32 | 3 | 41 | 47 | 12 | 41 | 15 | 44 | 100 | 0 | 0 | 65 | 12 | 24 | 85 | 15 | 0 | NA | NA | NA |
| Gee (2016) | 50 | 50 | 0 | 50 | 50 | 0 | 0 | 67 | 33 | NA | NA | NA | 33 | 0 | 67 | NA | NA | NA | 67 | 33 | 0 |
| Linardon (2020) | 63 | 37 | 0 | 22 | 70 | 7 | NA | NA | NA | NA | NA | NA | 48 | 0 | 52 | NA | NA | NA | NA | NA | NA |
| Senanayake (2015) | 100 | 0 | 0 | 29 | 57 | 14 | 29 | 71 | 0 | 57 | 43 | 0 | NA | NA | NA | NA | NA | NA | NA | NA | NA |
| Weisel (2019) | 75 | 6 | 19 | 44 | 50 | 6 | 38 | 6 | 56 | NA | NA | NA | 62 | 25 | 12 | 38 | 56 | 6 | NA | NA | NA |
| Whittaker (2016) | 92 | 8 | 0 | 92 | 8 | 0 | NA | NA | NA | NA | NA | NA | 75 | 8 | 17 | NA | NA | NA | 8 | 83 | 8 |
| Whittaker (2019) | 94 | 6 | 0 | 75 | 25 | 0 | NA | NA | NA | 100 | 0 | 0 | 88 | 6 | 6 | NA | NA | NA | NA | NA | NA |

Risk of bias summary aggregated across meta-analyses. Random Seq = random sequence generation; Allocat Conceal = allocation concealment; Blind Person/Partic = blinding of personnel and participants; Blind Outcome = blinding of outcome assessor; Incomp Data = incomplete outcome data; Select Report = selective reporting; Other = other bias; + = low risk of bias; ? = unclear risk of bias; - = high risk of bias.
