# Supplementary material for: Mobile phone-based interventions for mental health: A systematic meta-review of 14 meta-analyses of randomized controlled trials
Source: PLOS Digit Health. 2022 Jan 18;1(1):e0000002. doi: 10.1371/journal.pdig.0000002 (PMC8881800; doi:10.1371/journal.pdig.0000002)
Supplement: S1 Fig — (DOCX) [file pdig.0000002.s002.docx]

**Supplemental Materials Figure S1**. PRISMA flow diagram
