# Supplementary material for: Mobile phone-based interventions for mental health: A systematic meta-review of 14 meta-analyses of randomized controlled trials
Source: PLOS Digit Health. 2022 Jan 18;1(1):e0000002. doi: 10.1371/journal.pdig.0000002 (PMC8881800; doi:10.1371/journal.pdig.0000002)
Supplement: Attachment — Submitted filename: Response to reviewer comments.docx [file pdig.0000002.s005.docx]

Editor:

1. Please amend detailed Financial Disclosure statement. This is published with the article, therefore should be completed in full sentences and contain the exact wording you wish to be published. State what role the funders took in the study. If the funders had no role in your study, please state: “The funders had no role in study design, data collection and analysis, decision to publish, or preparation of the manuscript.”

RESPONSE: This change has been made.

2. Please provide separate figure files in .tif or .eps format only, and remove any figures embedded in your manuscript file. If you are using LaTeX, you do not need to remove embedded figures.

RESPONSE: This change has been made.

3. We have noticed that you have uploaded supporting information but you have not included a list of legends.  Please add a full list of legends for all supporting information files (including figures, table and data files) after the references list.

RESPONSE: This change has been made.

4. We notice that your supplementary figures and tables are included in the manuscript file. Please remove them and upload them with the file type 'Supporting Information'. Please ensure that all Supporting Information files are included correctly and that each one has a legend listed in the manuscript after the references list.

RESPONSE: This change has been made.

5. We have now received two reviews on your recent submission. We would like to invite you to undertake the major revisions, as outlined by the reviewers. Once this is complete, we will be able to make a decision on your paper.

RESPONSE: Thank you for the opportunity to revise our manuscript. We appreciate the helpful comments the reviewers have provided.

 Reviewer #1:

1. Given the extensive availability of mobile phone-based interventions available to consumers of mental health services, a synthesis of the literature to determine their efficacy is required. This manuscript is well written, uses an appropriate methodology, and draws conclusions consistent with the data presented. I believe significant clarity regarding the terminology used is required. This will improve clarity in regard to the methodology, particularly related to categorisation of conditions for each PICO category, and the data synthesis. Please find specific comments related to each section of the manuscript below.

RESPONSE: Thank you for your comments. We hope the changes we have made in response adequately address your concerns.

2. Introduction: The introduction is well written, reviews the relevant background literature and presents a clear purpose. The following comments are to add clarity.

RESPONSE: Thank you for these suggestions.

3. Please define mobile phone-based interventions so it is clear to the reader the types of interventions it includes and use the term consistently. For example, mobile mental health interventions and mobile health interventions also seem to be used but could refer to wearable devices, which are not included in this review.

RESPONSE: We now define mobile phone-based interventions as specified in our preregistration on pp. 3-4.

4. P4, regarding references 18, 19, 20, suggest referring to these as systematic reviews rather than reviews, to distinguish between them and meta-analyses.

RESPONSE: This change has been made.

5. P5, please provide more detail regarding the Lecomte et al meta-review. Limitations have been identified but it would benefit to reader to understand why the stated limitations might have confounded that study’s conclusions and why the results of the current review might be more valid. To this end, it might help to explain what specifically this review can offer beyond the Lecomte paper.

RESPONSE: We have expanded our discussion of the Lecomte et al. meta-review on pp. 5-6.

6. P4-5, I understand the argument that meta-analyses each define PICO differently, which might account for variation in outcomes between meta-analyses as they are essentially asking slightly different research questions. Are the last para on p4 and the second para on p5 saying the same thing?

RESPONSE: These paragraphs are making similar points. However, our intention in the earlier paragraph was to introduce the notion that meta-analyses do not fully agree, then to introduce meta-review as a means for deriving literature-wide conclusions, then to review the only available meta-review of this literature (Lecomte et al.), and lastly to get into details about why the primary meta-analyses may contradict one another and how a current meta-review can help resolves these discrepancies.

7. Could more detail be provided regarding the aims of the study? Particularly in regard to what is meant by ‘across PICO categories’. To do this, it may help to provide a very brief description of the method use to achieve this.

RESPONSE: We have provided more details regarding the aims of the study, what is meant by PICO categories, and a brief description of the methods we used on p. 7.

8. Thank you for describing the protocol deviations.

RESPONSE: We are glad this was helpful.

9. Coding of PICO subcategories - comparison conditions. Could the distinction between active, specific and non-specific be clarified? Providing specific examples that include both the intervention and the comparison condition might help (e.g., CBT smartphone app with a comparator of attentional control unlikely to have a therapeutic effect was coded as a non-specific control). I think part of my confusion is that attentional control is used as an example for active and non-specific categories. Could it also be explained why an active category is required, when you have a specific and non-specific active category?

RESPONSE: Thank you for highlighting this. As the reviewer noted, the active category includes a combination of specific and non-specific controls. We included all three of these categories (active, non-specific, specific) in order to most fully characterize the effect sizes reported in the primary meta-analyses. As meta-analyses coded control conditions differently, this seemed to us the best solution for representing the available literature. We have expanded our discussion of control condition types on p. 10. We also reiterate these distinctions in the note for Table 2 on p. 34.

10. Coding of PICO subcategories – intervention. Could the distinction between smartphone app, mobile phoned-based intervention and smartphone interventions be clarified? These terms may need to be revised as ‘mobile phone-based interventions’ seem to be used in the title and intro as an umbrella term capturing all types of mobile phone interventions, but in the method refers to a specific category of interventions.

RESPONSE: We agree this also should be clarified. We now define these categories more extensively on pp. 9-10. Of note, the term “mobile phone-based interventions” is used intentionally as an umbrella category in the introduction as well is in the results for effect sizes that are based on a combination of interventions that do not neatly fit into any of the other categories (e.g., combination of text message and smartphone app-based interventions; Whittaker et al., 2016).

Whittaker, R., McRobbie, H., Bullen, C., Rodgers, A., & Gu, Y. (2016). Mobile phone‐based interventions for smoking cessation. Cochrane Database of Systematic Reviews, 4, CD006611. doi: 10.1002/14651858.CD006611.pub4

11. Data synthesis - Could justification of why a representative ES for each unique combination of PICO was required when more than one ES for that unique combination was available? I would have thought that comparisons of these outcomes would be particularly valuable (and rare) in helping to determine homogeneity of results when using the same PICO criteria.

RESPONSE: We also had discussion on our team regarding whether it was preferrable to follow previous umbrella review studies which have provided a single representative effect size versus those which have tried to capture the range of reported effect sizes. In the end, it seemed most practically useful to provide the reader with a single effect size that was most likely to represent a particular combination of PICOs. Given the large number of combinations being reviewed, we were concerned that reporting all effect sizes would overwhelm the reader and muddy the depiction of the literature. Bear in mind that multiple effect sizes may have been available from across studies and/or within studies. For example, Cox et al. (2020) report eight distinct effect sizes, but many of the effect sizes seemed to use less relevant for evaluating the effect of text message-based interventions versus non-specific controls on depression (e.g., effect when the Beck Depression Inventory was used as an outcome measure, effect when various standardized depression measures were used, etc.). From our perspective, choosing a single representative effect size based on in our view defensible objective grounds (i.e., the effect size based on the largest sample) allowed us to avoid reporting many overlapping effect sizes and/or having to arbitrarily choose a single effect size to represent a particular PICO combination.

12. Results of individual studies - Similar to point 9, it is not clear to me why all the available ES are not reported here. I understand the value of presenting a ‘representative’ ES to report on the umbrella methodology category, but it seems valuable to also assess consistency of this outcome if the data is available. I concede, inconsistency may be due to variations in sample size, but potentially other study attributes, such as included study quality, may also contribute.

RESPONSE: Again, we appreciate this point and had similar discussions on our team. As noted, we were concerned that comprehensively reporting all effect sizes included in the eligible studies would have muddied rather than clarified the associations we were interested in. In some ways, our choice to select a single representative effect size on empirical grounds is most similar to how effect sizes are typically derived in meta-analysis – that is, authors report a single effect size for a single association and would rarely (without good reason, such as in a sensitivity analysis) arbitrarily split up the sample of studies and report multiple effect sizes based on partially overlapping subsamples.

13. Discussion: Conclusions drawn are appropriate and limitations included.

RESPONSE: Thank you.

14. Were any PICO combinations missing that if evaluated would be valuable contributions to the literature?

RESPONSE: This is an excellent question. Of course, given the many potential PICO combination, many were ultimately missing. We now highlight on pp. 22-23 several which may be particularly helpful to clarify through future randomized trials and meta-analyses.

15. P17, I understand why ES diminish as the comparison condition becomes more rigorous, but could an explanation be provided regarding why the evidence becomes weaker, i.e., is it specifically because there are too few meta-analyses (or RCTs) employing rigorous comparison conditions?

RESPONSE: We appreciate this question and have clarified on p. 18 that we are specifically referring to the grading of the evidence. As discussed on p. 11, this grading is impacted by the number of studies and the sample sizes within those studies (which impact the size of the confidence interval and associated p-value) as well as the degree of heterogeneity.

16. P16 and P17, I believe it would be useful to elaborate on the point that when the comparison condition is a specific active control (rigorous comparison condition) evidence showing non-superiority or a small ES is a not bad outcome, suggesting efficacy is at least equal to other therapeutic interventions.

RESPONSE: We appreciate this suggestion and have elaborated on this point on p. 19. In short, there is a large body of meta-analytic literature suggesting that psychotherapies that are intended to be therapeutic tend to perform equally well with the upper bound of differences between treatments estimated to be d = 0.20 (see Wampold & Imel, 2015). Thus, meditation apps showing an effect size of 0.28 on depression over specific active controls is similar to or even above the maximum magnitude of differences one might expect between two treatments.

17. It might be that safety is rarely reported because it is not considered important in mental health interventions. Could its importance be elaborated on in the discussion?

RESPONSE: We agree with the reviewer that safety is often not considered in mental health interventions. From our perspective, this is a known limitation of the broader psychotherapy literature that is only recently gaining prominence within the mobile health area. We have now included a recent review that confirms this on p. 20.

18. A potential limitation is that the interventions were not categorised in terms of the therapeutic model delivered (in addition to intervention quality), e.g., CBT, mindfulness-based CBT and so on. However, I understand given the amount of data being handled and the heterogeneity of RCTs may have made this task difficult.

RESPONSE: We agree this is an important limitation that can ideally be addressed in future meta-analyses (e.g., that test intervention type as a moderator). This is now discussed on p. 22. At once, it is worth acknowledging here that many apps are challenging to classify and there is as yet not a well-accepted taxonomy for these interventions.

Reviewer #2:

1. Overall, this is quite a neat paper, well written and thoughtfully constructed. While the initial objective to explore -in this systematic review - states that “mobile phone based interventions have been proposed as a means for reducing the burden of disease associated with mental illness”, this point could be more sufficiently explored/extended in this review. While the discussion section returns to this point, the method and results section do not provide enough evidence to come to this conclusion and -in my opinion - somewhat minimal in statistical evidence. Therefore this conclusion needs to be stated more cautious thereby openly discussing and acknowledging any limitations. The methods and subsequently results section could benefit from the inclusion of some more detailed information on the characteristics of the study characteristics (study outcomes, demographics, intervention type etcetera) of the meta-analysis, and subsequently these variables should be included in the analysis for the current review.

RESPONSE: Thank you for these many thoughtful comments. We respond below to your specific suggestions. We have tried to be responsive to these points, albeit within the limitations of the meta-analytic literature which we review. One broader point to clarify here is that the current study was not itself a meta-analysis, but rather was a systematic review of meta-analyses. Thus, we did not reconduct statistical tests reported in the primary meta-analyses but instead sought to depict the full scope of the meta-analytic literature via systematic review. Although we explicitly sought to include moderator tests that may have examined the characteristics noted by the reviewer (e.g., intervention type, demographics) only one study included moderator tests that were eligible for inclusion. We do agree that future meta-analyses could productively examine a wider range of study characteristics as factors that may account for heterogeneity in effect sizes. This point is noted on pp. 22-23.

2. It seems like the authors use two terms (EFFICACY and EFFECTIVENESS) to explain their objectives. As per my knowledge, the term EFFECTIVENESS is generally used to describe the results of RCTs in an ideal or controlled environment (laboratory). The term EFFECTIVENESS is more commonly used to describe the effect of an intervention in a natural environment or under real-world conditions. Since the selected RCTs in the meta-analyses included were all conducted in a controlled environment, I am confused by the use of these two terms throughout the manuscript. See also reference to efficacy in the Abstract but throughout the rest of the manuscript effectiveness is discussed. What is the justification for the use of these two terms? To be clarified and/or justified.

RESPONSE: We appreciate this point. We have now included brief definitions of both terms where they first appear (pp. 4-5). From our perspective, many RCTs of mobile phone-based interventions fall somewhere in a gray area between ideal / controlled conditions and real-world conditions. RCTs of unguided smartphone apps may be conducted with very minimal staff contact (e.g., Arean et al., 2016), thus reflecting the real world. In contrast, other RCTs occur in more tightly controlled environments (e.g., Bucci et al., 2018). However, in response to this comment we have modified our terminology and now discuss “efficacy” rather than “effectiveness” given that the included studies may or may not have involved additional research staff or other involvement that more closely mirror laboratory conditions than the real world. In other words, it seems more conservative to claim these represent efficacy estimates rather than effectiveness estimates intentionally designed to assess real world implementation.

Arean PA, Hallgren KA, Jordan JT, Gazzaley A, Atkins DC, Heagerty PJ, Anguera JA. The use and effectiveness of mobile apps for depression: results from a fully remote clinical trial. Journal of medical Internet research. 2016;18(12):e330.

Bucci S, Barrowclough C, Ainsworth J, Machin M, Morris R, Berry K, Emsley R, Lewis S, Edge D, Buchan I, Haddock G. Actissist: proof-of-concept trial of a theory-driven digital intervention for psychosis. Schizophrenia bulletin. 2018 Aug 20;44(5):1070-80.

3. Although in the first paragraph of the Introduction an effort has been made to explain the growing interest in mobile mental health interventions, this section could benefit from some restructuring, thereby elaborating on research results about the use of smartphone apps across the specific outcome categories, instead of referring to smartphone apps for mental health illness in general (especially since the results are presented as per condition). Adopting a neutral point of view, there is lots of research available examining the use of smartphone applications in the treatment/prevention of specific mental health disorders, that could be discussed here.

RESPONSE: Thank you for these suggestions. While we can appreciate how it may be useful to discuss the evidence by outcome category, from our perspective this evidence is largely unclear. This was, of course, precisely what motivated this meta-review. Thus, it seems most appropriate to us to keep the introduction focused on the general pattern of findings pertaining to mobile phone-based interventions for mental health and the discrepancy in interpretations of this literature, rather than detailing the patchwork of findings drawn from previous studies which we hope our study summarizes in a much more systematic and satisfying way.

4. Also reconsider to reference to smoking; alcohol/drug misuse to explain the burden of disease associated with mental health issues - not always appropriate.

RESPONSE: We believe the reviewer is raising the important question of whether smoking can reasonably be included in the burden of disease associated with alcohol and drug use. While we agree that smoking differs in important ways from other substances of abuse, nicotine use disorder is nonetheless categorized as a substance use disorder within the DSM-5. Thus, it seems important to consider when evaluating the impact of mobile phone-based interventions on mental health.

5. Furthermore, although important to emphasize, I am furthermore wondering if the detailed introduction of the use of meta-reviews can be shortened.

RESPONSE: Thank you for this suggestion. As we do think a thorough introduction of meta-reviews will be helpful for readers less familiar with this method, we have retained this content.

6. P4 'While the first generation of these studies focused on feasibility and acceptability, the accumulating evidence clearly indicates that people suffering from all mental health conditions, including even schizophrenia, are interested in and willing to use technology as part of their care'

   It would be helpful to explain why the reference to schizophrenia was made here.

RESPONSE: Thank you for this question. We note schizophrenia specifically as from our perspective it supports that notion of wide acceptability and openness to mobile phone-based interventions. Given schizophrenia can be linked with severe disruptions in behavior, cognition, interpersonal relationships, and activities of daily living, evidence for feasibility and acceptability in a group that on average experiences considerably more disability than the general population speaks to the potential for these interventions for large swaths of the population, not simply those with very mild psychiatric symptoms. This is now noted on p. 4.

7. With respect to the eligibility criteria (p7 and elsewhere) and when presenting the results in Table 1/2, it would be helpful to indicate some more detailed information about the interventions examined in the included studies (i.e. interventions of preventative nature; behavioral etcetera) and use type of intervention as a comparison between studies, in addition to the use of the current categorization of interventions (i.e. text messaging, smartphone intervention, meditation apps etcetera) . This should be taken into account when analysis the results and could potentially influence the results found.

RESPONSE: We appreciate this point and fully agree that there could be many valuable ways to split the sample of studies. And, as noted by the reviewer, these could be used to predict variation in effect sizes (i.e., tested as statistical moderators). However, as we were not conducting a primary meta-analysis but instead conducted a systematic review of primary meta-analyses, we are restricted to the groupings and moderator tests available in the literature. We have attempted to strike an optimal balance between characterizing details of the primary meta-analyses (e.g., including information on key PICO elements) while avoiding overly detailed description of factors that varied within primary meta-analysis (e.g., we did not report the various kinds of interventions that each meta-analysis included as this would have generated very long and we believe difficult to follow tables and results). Nonetheless, we fully agree with the reviewer that these are precisely the types of characteristics that would be valuable to examine as moderators in future primary meta-analyses. This is noted on pp. 22-23.

8. In the Methods and Results it furthermore doesn't mention how clinical condition was determined for each of the samples in the included studies (definition of clinical diagnosis; subclinical/clinical). It would be helpful if this was clarified further.

RESPONSE: We appreciate this comment. As with other study characteristics, we followed definitions used in the primary meta-analyses. This is now described in greater detail on p. 9.

9. P7. Databases were searched since inception until October 31st, 2020. Would be worth to update search to a more recent date.

RESPONSE: We appreciate the reviewer raising this point. Of course, searches can always be updated for systematic reviews and meta-analyses. We opted not to update the search at this time for two reasons. First, from our perspective, the search date remains within a reasonable range. Searching along with initial abstract review and screening often takes a month; coding, analyzing, and writing typically takes 2-3 months; and the peer-review process itself can take several months. Second, and more importantly, the study in its current form includes a sufficiently large number of meta-analyses (k = 14) and primary RCTs (k = 145) that it seems likely to accurately represent the state of knowledge, particularly given meta-analyses accumulate more slowly than primary RCTs.

10. P7/P8 - In the initial search for relevant meta-analysis the search terms digital health and e-health weren't included? Consider updating search terms when exploring recent publications.

RESPONSE: We spent considerable time as a team determining the optimal set of search terms. Ultimately, we felt that our finalized list adequately covered the literature. Of course, there are always additional terms that might have been considered, particularly given that the mobile health literature has used a wide variety of terms. In our view, it seems unlikely that an eligible meta-analysis studying mobile phone-based interventions would not have included one of the technology-related terms we included (e.g., “mobile phone,” “smartphone,” etc.). As with updating the search date, this suggestion would delay the publication of our findings which we believe accurately reflect a robust evaluation of the available literature.

11. The authors should comment on whether or not the raters conducted screening/rating/data extraction independently AND blindly (p8). It would be helpful if this was clarified further.

RESPONSE: We have clarified on p. 9 that screening for inclusion and rating for quality was conducted independently and blindly. Study- and effect size-level characteristics were coded independently but not blindly.

12. To be able to summarize the includes studies based upon the PICO principles population and condition was coded. Sample population was coded. It would be helpful to clarify coding adults vs adolescents and how the clinical condition coding was established -this is important to know in the interpretation of the results. I would furthermore suggest to include more information about the age distribution across studies and include this in the Results section and Tables.

RESPONSE: We appreciate this suggestion and have added details about how PICO categories were coded. As our study is a review of reviews, much of this coding relied on decisions that were made at the primary meta-analysis level. That is, we generally did not code primary RCT-level characteristics for RCTs included in each meta-analysis but rather sought to characterize the meta-analysis-level characteristics. While it would be possible to report a range of mean ages across the RCTs included in each meta-analysis, we feel this level of detail will distract from the meta-analysis-level points we are seeking to make (and, of course, the RCT-level mean ages themselves represent a summary statistic for a within-RCT distribution of ages) and will not add much to readers’ ability to interpret the literature-wide patterns. Thus, it seems to us that simply characterizing whether the included RCTs were focused on adult, non-adult, or mixed adult and non-adult samples is most parsimonious and useful for the reader.

13. Summary Measures P9. It is stated that "Standardized mean difference (i.e., Cohen’s d, Hedges’ g) served as our primary effect' - So both Cohen's and hedges are reported? Or all effect sizes converted to either hedges/cohen? I am assuming the latter happened for subsequent analysis but should be clarified. For example, Figure 2 states cohen but unclear for ES in Table 2. It is furthermore unclear when effect sizes to be considered as not-eligible effect sizes to be included in this review.

RESPONSE: We appreciate this question. Again, as we were summarizing at the meta-analysis-level (rather than conducting a primary meta-analysis ourselves), we used whatever effect size the primary meta-analysis authors included. Thus, the effect sizes may have been Cohen’s d or Hedges’ g. As both effect sizes are in standardized mean difference units, we refer to them as Cohen’s d for simplicity. This is now clarified on p. 11. We converted alternative standardized effect sizes such as odds ratios into Cohen’s d units. We have also clarified on p. 8 that effect sizes were considered ineligible if they were based on a combination of comparison condition types (e.g., combined specific active controls and inactive controls).

14. Synthesis the Results P10. I could not find any information about time points outcomes assessed? What about variations in follow-up period? Are effect sizes corrected for that? Would be helpful to discuss this in the Methods and Results.

RESPONSE: Thank you for raising this question. In the primary meta-analyses that were included, effect sizes generally reflected post-treatment, which is now noted on p. 13. One complication is that many mobile phone-based interventions are by their nature potentially long lasting (e.g., access to a smartphone app can be ongoing). Thus, the typical notion of “follow-up” from RCTs of time-limited interventions does not entirely seem to map onto the mobile phone-based intervention literature. This is now discussed on pp. 23-24. One point of clarification to state again here is that we did not conduct a primary meta-analysis ourselves and thus did not make primary decisions about how to handle treatment of the length of follow-up. Given our use of a meta-review methodology, we relied on the effect sizes as reported in the primary meta-analyses.

15. P11 - Risk of bias Within Studies. It would be good to have the results of Supplemental Materials Table 1 explained a little more here - consider Eggers regression test or presenting p values.

RESPONSE: We appreciate this suggestion. We have summarized what we believe are the key findings represented in Supplemental Materials Table 1 in our discussion of Risk of Bias Within Studies on pp. 13-14. We include the study-level information for interested readers who may wish to examine this finer-grained detail for themselves. However, we do not believe that additional discussion is warranted or would add to the general readers’ understanding of our study or this literature. In terms of the Egger’s regression test, here again it is important to keep in mind that we conducted a meta-review of meta-analyses rather than a primary meta-analysis ourselves. Thus, our discussion of risk of bias within and between studies is based on information reported in the primary meta-analyses rather than analyses conducted by us. From our perspective, evaluating reporting of risk of bias and other meta-analysis-level characteristics and methodologies across the literature can itself be quite valuable for guiding future meta-analytic and primary RCT work in this area.

16. In line with my previous comments, Table 1 includes a limited amount of information about each meta-analysis. I think more information about demographics where possible (such as age range, gender), clinical conditions, what instruments used to measures outcomes, as well as characteristics of the interventions, should be included in order to permit a more in depth critical appraisal of the studies and inform discussions with respect to the representativeness of the findings and draw any conclusions about effectiveness of the smartphone 'intervention' examined.

RESPONSE: As noted above, our review focused on meta-analyses rather that primary RCTs included in the respective meta-analyses. While it would be theoretically possible to code primary RCT-level information for the 145 included studies, in our view this information would muddy rather than clarify the literature we are hoping to summarize which is the meta-analytic rather than the primary RCT literature. We certainly agree that large meta-analyses have an important place (and indeed we have conducted some of these meta-analyses ourselves). At once, we also believe that reviewing the meta-analytic literature (rather than the primary RCT literature) is a valuable method for depicting literature-wide trends.

17. Please furthermore ensure the index of Table 2 explains all abbreviations included in the Table.

RESPONSE: We have added descriptions of all abbreviations in Table 2 on p. 34. In addition, for clarity, we now also define the coding of control conditions and strength of evidence in the table note.

18. A PRISMA checklist should be included as supplementary material or as an appendix.

RESPONSE: This change has been made.
